# Varicocele in the Male

**Published:** 1911-02

**Authors:** Aime Paul Heineck

**Affiliations:** Surgeon to the Grace, Reliance and Cook County Hospitals; Senior Prof. of Surgery, Reliance Med. Coll.; Adj. Prof. of Clinic Surgery, Coll. of Physicians and Surgeons, University of Ills., Chicago, Ills.


### VARICOCELE IN THE MALE.

*(a) Discussion of the Condition. (b) A New Operative Procedure for Its Successful Treatment.*

BY AIME PAUL HEINECK, M. D.

*Surgeon to the Grace, Reliance and Cook County Hospitals; Senior Prof. of Surgery, Reliance Med. Coll.; Adj. Prof. of Clinic Surgery, Coll. of Physicians and Surgeons, University of Ills., Chicago, Ills.*

From the standpoint of scientific accuracy and completeness, the literature that has been published relative to varicocele is notoriously unsatisfactory. Despite the frequency of this pathological and clinical condition, our knowledge concerning its significance, its etiology, its pathology, and the results of its treatment by operative measures is honeycombed with deficiencies. The purpose of this article is to stimulate and to facilitate the efforts of those that might feel impelled to elucidate some of the many as yet unsolved points of this definite anatomical and clinical entity.

At the Cook County Hospital, from January, 1906 to July 1st, 1910, inclusive, 155 cases of varicocele were subjected to operation. At least as many other patients were refused operative relief. The operations performed were venous resection, scrotal resection or both combined. The youngest patient operated upon was eleven years old, the oldest 57 years. The ages of the patients are shown in the following table:

| | |
|---|---|
| 11-20 years | 36 patients |
| 21-30 years | 77 patients |
| 31-40 years | 18 patients |
| 41-50 years | 10 patients |
| 51-60 years | 3 patients |

Age not ascertained in eleven cases.

In 4 cases, the affection was right-sided; in 10, bilateral; and left-sided in 131 cases; in 10 cases, the side affected is not recorded. Six cases were associated with an inguinal hernia of the same side, and four with hemorrhoids. In a few cases, the presence of varicose veins of the leg is noted. Though the institution admits individuals of all races, not one of the patients operated upon was colored.

Quain (1), defines varicocele as follows: "A dilated, elongated and tortuous condition of the veins of the spermatic cord, due either to increased pressure within the vessels or to diminished resistance in the walls of the vessels and in the surrounding structures." The pathological dilatation lengthening, and tortuosity are limited almost always to the spermatic vein and its branches. Exceptionally, the cremasteric and deferential veins and their branches participate in the process. The veins of the scrotum may also show varicose dilatations. The spermatic vein originates at the posterior border of the testis as a thick closely woven network and forms the pampiniform plexus. This plexus consists of from eight to ten veins most of which lie anterior to the vas deferens; it passes upward, enters into the formation of the spermatic cord, courses through the inguinal canal and finally forms a single trunk in the abdominal cavity. In varicocele, the venous lengthening, tortuosity and dilatation are permanent and are associated with histo-anatomical changes in the vessel walls. Temporary dilatation, such as compression of short duration can determine, and which disappears completely after the removal of the compressing agent is not varicocele.

Varicocele may be unilateral or bilateral (2), may be primary or secondary, that is, idiopathic or symptomatic, may be complicated by the co-existence of other local pathological states: hernia, vaginal hydrocele, tumors of spermatic cord, etc., may be associated with a fully developed or with an undeveloped testicle. (3). In Gould's case (3), the testicles were small but not wasted. The following varieties are recognized:

(a). Simple dilatation and varicosity of the veins with or without slight scrotal relaxation.

(b.) Orchidoptosis.

(c.) Varicosities and orchidoptosis.

All authors state that the left side only is involved in by far the larger number of cases (80% to 90%, Chassaignac, Dardignac (4, 5), 92%, Istomin (5a). Clinical observation amply confirms this statement

Statistics are not in accord as to the frequency of the condition. Senn (2), in 9,815 recruits examined, found varicocele present 2,078 times, that is, in 21.17%. In 15 of these cases, the affection occurred on the right side; in 17, it was bilateral; in the remaining cases, the left spermatic cord was the seat of trouble. French military commissions report varicocele as occurring in 6.4% of all recruits. No age is exempt. Though it occurs at all ages it is rare both in the young ( a and b) and in the old. Gould (3), had a case of varicocele occurring in a boy four years old, and another case in a boy eleven years old. Its period of greatest incidence is between the ages of 20 and 40. Landouzy gives the fellowing table:

In 13 cases the varicocele was first noted between 9 and 15 years.

In 29 cases the varicocele was first noted between 15 and 25 years.

In 3 cases the varicocele was first noted between 25 and 35 years.

Curling (7), gives the following statistics:

2 cases were between 10 and 15 years when they came under medical notice.

26 cases were between 15 and 25 years when they came under medical notice.

14 cases were between 25 and 30 years when they came under medical notice.

5 cases were between 35 and 45 years when they came under medical notice.

3 cases were between 45 and 65 years when they came under medical notice.

No race is immune. It has, however, been observed that negroes are practically free from varicocele. In them, the scrotum is close fitting and less lax than in Caucasians.

An intelligent understanding of the condition and of its treatment is aided by a correct knowledge of the regional anatomy of the spermatic cord and of the scrotum.

In idiopathic varicocele, the patients frequently complain of a sense of weight and of a dragging pain in the scrotum and groin, relieved on lying down and increased by severe bodily strain. One must not forget that an entire absence of subjective symptoms is not uncommon and that there are varicocele of large size which produce no subjective symptoms whatever, no pain, no sexual debility, no wasting of the testicle. In idiopathic varicocele, the veins collapse when the patients assume the horizontal posture. In all types of varicocele, actual or imaginary, the morbid tendencies are frequently aggravated by quack advertisements, commercial medical literature and the artful suggestions of the charlatans. (4.).

The symptomatic type of varicocele is almost invariably painless. One of its characteristics is that the veins remain distended when the patient assumes the reclining posture.

The secondary or symptomatic type of varicocele may be caused:

1. By neoplasms of the kidney. In 16 cases of renal tumors, six had determined a symptomatic varicocele (8.) Reclus' (9), patient, an elderly man, who presented a right-sided varicocele consecutive to a renal cancer.

2. By occlusion of the left renal or of either spermatic vein by a neoplastic growth. In Hochenegg's case (8), the symptomatic varicocele was due to the invasion and obstruction of the left renal vein by the renal growth.

3. By compression of the spermatic vein exerted by cancerous lymphatic glands or by renal tumors, by enlarged retroperitoneal glands. Delbet's patient (10), was 57 years of age and complained of a well-marked, but painless, right-sided varicocele, which had developed without apparent appreciable cause and had increased progressively in size. The autopsy showed that a cancerous juxta-pancreatic lymphatic gland had by compressing the spermatic vein determined the venous ectasia.

4. By kinking of the spermatic vein, due to inflammatory adhesions, to the weight of tumors, to prolapse of the left kidney, etc.

Among the many causes, all more or less inadequate, advanced as predisposing, contributory or exciting factors to the production of idiopathic varicocele, the following are the most frequently cited:

1. The great length, the vertical course, the dependent position and the great tortuosity and the frequent anastomosis of the spermatic veins.

2. The abnormal thinness of the vein walls.

3. The almost complete absence of support afforded the spermatic veins by the loose tissue which surrounds them.

4. The pressure exerted by the contraction of the abdominal muscles upon the spermatic veins as they course through the inguinal canal, as by straining at stool, etc., 1, 2, 3, 4, 5, are anatomical conditions common to all healthy men.

5. The plexiform arrangement of the spermatic veins in the scrotum and their termination in one small vein in the abdomen. The radicles of the spermatic veins emerge from the back of the testis, receive tributaries from the epididymis, unite and form a convoluted plexus called the spermatic plexus (plexus pampiniformis). Combined lumen of the veins is large as compared with that of artery (spermatic artery), so that the vis a tergo is reduced to a minimum (15c).

6. Aplasia (11, 12), predominating in the veins and valves thereof. "Varicocele is a congenital aplasia of the veins of the spermatic cord." (Escat.) "Varicocele is a genito-crural fibro-muscular aplasia, chiefly affecting the left side." (Longuet.) "Varicocele consists in a loss of tone of all the genito-scrotal tissues." (Longuet.)

7. The absence in the diseased spermatic veins of efficient valves. The minor frequency of right-sided varicocele is partly due to the almost constant presence of an efficient valve at the point where the right spermatic vein debouches in the inferior vena cava. "These veins are provided with valves, but occasionally the valve at the orifice of the left spermatic artery is absent." (Cunningham's Anatomy.)

8. Anything which tends to obstruct the free return of blood through the spermatic veins from the testis, as, for instance, fecal masses in the caecum or in the sigmoid colon, pressing upon the spermatic veins.

9. Undue activity of the sexual apparatus. In many individuals, sexual fatigue is accompanied by a considerable relaxation of the scrotal tissues. In warm climats, lengthening and relaxation of scrotum is an almost invariable accompaniment of varicocele. (14, 25.)

10. Occupations exposing the scrotum to frequent slight traumatisms (horseback riding), and also such as necessitate continuous and prolonged standing. Varicocele is not uncommonly met in those who are long in the saddle and also in those who ride the bicycle to excess.

11. Heredity, traumatisms, previous inflammatory states and other indefinite factors.

The reasons advanced to explain the great preponderance of left-sided varicocele are not convincing:

1. Inferior muscular development of the left side of the body from predominant use of the right.

2. Of the two spermatic veins, the left vein is the longer. Schultz says that the right spermatic veins outlet is 1 1-2 inch lower than the left. The difference in length of the two veins is slight and does not exceed that between the two iliac veins, which latter has not led to a similar disproportion in the occurrence of varicosities in the veins of the lower extremities. (Gould, 3.)

3. The left spermatic vein is exposed to being compressed by a sigmoid colon loaded with fecal matter. Constipation is not more frequent in those that have varicocele than in other individuals of the same age and class. Constipation is frequent in old men; varicocele is rare in them. Those that look upon constipation as a cause of varicocele find it difficult to explain why the veins collapse instead of becoming turgid upon the assumption by the patient of the recumbent position.

4. Rectangular implication of the left spermatic vein into the left renal vein.

Varicocele (15a), of the broad ligament, a condition in the female that bears some analogy to varicocele in the male, is also of more frequent occurrence on the left side. Kanavel and Miller say: "It is to be noted that of twelve cases of primary varicocele of the broad ligament, six occurring upon the left side alone, in six it was bilateral, in no case occurring upon the right side alone." (15b.) Authors have sought to explain the greater frequency of left-sided varicoele of the broad ligament by the same reasons that are advanced to account for the more frequent occurrence of left-sided varicocele of the spermatic cord (15c).

In the differential diagnosis of varicocele, one only needs to consider hernia, lipoma, hydrocele communicans. Varicocele may be confounded with an epiplocele because both have a cord-like arrangement.

### Treatment.

If every case of varicocele is operated on indiscriminately, a fair percentage of patients will suffer permanent bodily harm, locally in the testis, and generally in body and mind (13 a and b). It is a matter of general knowledge that many varicocele operations are performed in the absence of positive indications. Charlatans have found it very lucrative to needlessly operate cases of imaginary varicocele and cases of very slight dilatation of the branches of the spermatic vein. One cannot too strongly condemn the subjecting of a patient to a needless operation.

In the treatment of varicocele operative surgery has a legitimate and well-defined sphere of action. In this, as well as in other surgical conditions, we consider it important that operative indications and contra-indications be formulated with precision.

We are of the opinion that operative intervention is absolutely contra-indicated and not possible:

1 In pseudo-varicocele. When the veins of the spermatic cord are not the seat of lesions demonstrable to inspection or to palpatation, a varicocele is not present. The surgeon must not accede to the importunities, to the requests of hypochondriacs and the neurasthenics, who insist on being operated upon for an imaginary varicocele. Many of these individuals are hardened, pessimistic and dangerous neuropaths (16). Owing to the fact that in these cases there is not any vein lesion present, surgical intervention does not benefit the existent orchialgia, testicular neuralgia, or other symptoms of which these patients complain.

2. In symptomatic varicocele. The cure of a symptomatic varicocele is dependent almost entirely upon the surgeon's ability to remove the causative factor.

3. In varicocele occurring in individuals suffering from constitutional states that forbid the performance of operations of election, even if such operations of choice do not entail risks. The various operations performed for the relief of varicocele are without danger to life. Among unfavorable constitutional states, the most important are malignant disease, diabetus mellitus, advanced renal, cardiac and hepatic affections, etc.

### Indications for Operation.

Relief by operative means is indicated in all cases of varicocele:

1. In which there co-exists an inguinal hernia of the same side, be the hernia complete or incomplete, reducible or irreducible, an enterocele, an epiplocele, an entero-epiplocele. The pressure of an ill-fitting truss cannot only aggravate an existing varicocele, but can also lead to the development of this pathological state. If a hernia co-exists with a varicocele, the curative operation for the varicocele is to be supplemented at the same sitting by one for the radical cure of the hernia. Carta (17a), in 150 cases of varicocele, found six co-existing with a hernia of the same side. In 21 patients, operated upon for varicocele, Narath (17b), found inguinal hernial sacs in five. In one patient, both the hernia and the varicocele were bilateral.

2. In which there co-exists on the same side a hydrocele (18), of the tunica vaginalis testis. Both conditions, varicocele and hydrocele, should be remedied at one and the same operative sitting. For the varicocele, the operation described at the close of the article should be performed. The hydrocele is best met by incising longitudinally the tunica vaginalis and everting it around the epididymis and scrotal portion of the cord. The upper margin of the everted tunica vaginalis is then sewed to the subpubic fibrous tissue. Carta (17a), in 150 cases of varicocele found in twenty cases a co-existing hydrocele of the tunica vaginalis testis of the same side.

3. In which there is present on the side an encysted hydrocele of the cord. The same incision gives access to both pathological states.

4. Associated with or dependent upon the presence of a tumor of the spermatic cord (19). In these cases, the surgeon is confronted by a double indication, the removal of the neoplasm and the correction of the varicocele.

5. Having a history of recurrent attacks of phlebitis and of thrombosis (Burghard, 20a, Longuet, 20b). Two of Narath's cases presented a lipoma of the cord. Here, the operation is preferably performed during a quiescent period; at other times, a troublesome spreading thrombosis may originate at the seat of ligation.

6. In which there has been an accidental or spontaneous rupture of one or more veins of the affected spermatic cord. The rarity of rupture is partly explained by the mobility of the spermatic cord, and by the elasticity of its various tunics which together enable the veins to easily shift away from traumatic insults. Patel (21), reports a case of co-existing hydrocele and varicocele of the same side, in which there occurred an apparently spontaneous rupture of one or of several veins of the varicocele. This rupture converted the hydrocele into a hydro-hematocele. Patel exposed and ligated the bleeding points, removed the extravasated blood, and subjected the hydrocele or hydro-hematocele to appropriate operative treatment. Rupture of a varicocele may prove fatal. A case of this nature is reported in the Lancet (22). The patient had a left-sided varicocele, as a consequence of a blow received on the left scrotum, the latter swelled to the size of a child's head. Incision of the scrotal swelling was followed by discharge of fresh blood, which continued to escape until the patient suddenly died. It was demonstrated that the uncontrolled and fatal hemorrhage resulted from traumatic rupture of a varicose vein of the spermatic cord.

7. That show more than a moderate degree of venous dilatation, because in these cases the functional integrity of the testis is either seriously menaced or involved. It is desirable to rid the patient of the disagreeable consciousness of the continual presence of a testicular tumor (Lydston, 29). In mild cases, without symptoms, operative treatment is not required. The patient's mental annoyance and exaggerated apprehensions must be allayed by sensible advice (Bennett). In a case reported by Loumeau (23), the patient was 18 years of age and presented for treatment a voluminous and painful varicocele extending downward as low as the middle of the thigh. Berger (23a), in one patient resected a spermatic vein, the calibre of which equalled that of the little finger.

8. That are productive of neuralgic pain in the testis, of pain radiating in the back and a characteristic dragging sensation. That is, in all types of painful varicocele, the painfulness of which is not controlled by the wearing of a well-fitting suspensory and the employment of judicious non-operative therapeutic measures. No constant relation exists between the size of the varicocele and the degree of pain and other subjective symptoms present. Not uncommonly the patient is more irritable than the varicocele is painful. Varicocelic pain recognizes various causes: compression of nerve-filaments by varicosed veins; neuritis, due to ectasia of the vaso-nervorum, atrophy of the gland, the patient's physical state, etc.

9. That are associated with serious nervous disturbances, such as neurasthenia, psyshic disorders, tendency to suicide, etc. (25.) These patients harassed by the presence of their varicocele, often develop a distressingly hypochrondriacal state of mind. The operation does not make the neurasthenia worse, but often improves the general state of the patient.

10. Showing a steady increase in size and progression of avoidance of constipation, cold ablutions of the parts, sexual hygiene, the wearing of a well-fitting suspensory, etc.

11. That show calcareous changes in the vessel-walls. Dardignac (5), and others, report cases of varicocele in which the markedly dilated veins were the seat of calcareous incrustations.

12. When the patient wishes to enter some public service, civil, police, military, or naval, and the varicocele is the only existent physical disqualification.

13. If disease of the opposite testicle be present, hydrocele, tuberculous epididymitis, cystic disease of the testis, etc. In the presence of disease of the opposite testis, it is important to preserve the functional and anatomical integrity of the unaffected testis. Le Fort, (26), presented to the Societie Centrale de Medecine du Nord a patient inflicted with a left-sided varicocele extending as far as the lower third of the thigh. The right testicle extended lower than the left, was hypertrophied and the seat of a hydrocele.

14. If the opposite testis is lost.

15. In which the nutrition of the testis is threatened. A varicocele can impair the nutrition of the testis in various ways: The process may extend to the intra-testicular veins, by its volume may injuriously compress the organ, the passive hyperemia of the gland may prove deleterious to latter's nutrition, etc. "When highly or rapidly developing, the dilatation of the veins interferes so much with the nutrition of the gland as to occasion wasting." Curling (7).

16. Associated with evident scrotal changes, marked pendulousness, profuse scrotal sweating and obstinate dermic lesions of the scrotum.

17. When the condition is bilateral.

It is our opinion that all the various subcutaneous operations for varicocele should be completely discarded. If a varicocele be of such a degree or nature as to necessitate operative relief, only such methods of treatment should be resorted to as are appropriate. The patients objections to an open operation should be surmounted or disregarded. One of the most manifest tendencies of modern surgery is to abandon all subcutaneous methods of operating, and among the subcutaneous methods of treatment that have fallen into almost complete disuse can be mentioned the injection treatment of goitre (27), the injection treatment of hernia, of vaginal hydrocele, Bottini's operation for prostatic hypertrophy, subcutaneous suturing of fractured patellae (28), etc. It is incontrovertible that the less an operator knows of anatomy and of surgical operative technique, the more reluctant he is to abandon subcutaneous methods of operating. (29.)

ADVANTAGES OF THE OPEN OPERATION FOR RESECTION OF VARICOSE SPERMATIC VEINS.

1. Under the guidance of the sense of sight, every step of the operation can be carried out with precision. Insufficient or excessive removal of veins does not occur. The operator removes only that amount of veins, the ablation of which cannot lead to undesirable immediate or remote sequelae. With the subcutaneous methods, the veins can be ligated, but not resected.

2. The inclusion of the vas deferens in the ligature can always be avoided. In the subcutaneous operations, a thickened vein may be isolated under the impression that it is the vas, and the vas be ligated with some of the varicose veins. The ligation of the vas deferens permanently occludes the excretory duct of the testicle of that side. From the standpoint of procreative power, the testicle whose vas has been ligated is and remains valueless.

3. More complete hemostasis is secured. With the open method, the complete control of hemorrhage is easily effected. In the course of the subcutaneous operations, a small or a large vessel may be accidentally punctured, such a puncture can lead to the formation of a hematoma, can give rise to an extravasation of blood into the scrotal tissues. Either of these accidents necessitates an incision of the scrotum, followed by evacuation of the extravasated blood, and ligation of the bleeding points.

4. A slight lengthening of the usual incision enables the surgeon to appropriately treat co-existing neighboring pathological states, as hernia, vaginal hydrocele, neoplasms of the spermatic cord, etc.

5. The simplicity of technique of the open operation places them within the reach of all operators.

The permanent occlusion or obliteration of the ligated, divided or undived spermatic veins, is effected by the organization of the thrombi that form on the proximal and distal sides of the ligatures placed on the non-divided vessels, or on the ligated proximal and distal ends of the divided vessels. In the subcutaneous methods, the expectation of permanent cure is also based on the organization of thrombi forming on either side of the ligatures. For the organization of a thrombus time is required. The transformation of a thrombus into a block of connective tissue is effected not by the cells contained in the thrombus, but by the proliferation of the cells of the injured vessel-wall. The vessels of the occluding block of connective tissue are derived from the vaso-vasorum of the ligated vein. Previous to the organization of the thrombi, undue activity on the patient's part may lead to the dislodgement or detachment of thrombotic particles, and to emboli formation and its consequences. Therefore, all forms of operative treatment that do not exact confinement of the patient to bed for at least a week are to be condemned. Early activity on the part of the patient has determined such unfortunate accidents as pulmonary infarcts. (30.)

*(To be Continued.)*

